# A new species of the genus *Acanthosaura* Gray, 1831 (Reptilia, Lacertilia, Agamidae) from the China-Myanmar border region in southwestern Yunnan Province, China

**DOI:** 10.3897/zookeys.1290.195408

**Published:** 2026-08-27

**Authors:** Kai Wang, Zhuo-Yu Lu, Gui-Gang Zhao, Wei Shi, Ru-Wen Wan, Na Zhu, Jie-Qiong Jin, Jing Che

**Affiliations:** 1 Yunnan Key Laboratory of Biodiversity and Ecological Conservation of Gaoligong Mountain, Kunming Institute of Zoology, Chinese Academy of Sciences, Kunming, 650223, China Yunnan Key Laboratory of Biodiversity and Ecological Conservation of Gaoligong Mountain, Kunming Institute of Zoology, Chinese Academy of Sciences Kunming China; 2 Southeast Asia Biodiversity Research Institute, Chinese Academy of Sciences, Yezin, 05282, Myanmar Southeast Asia Biodiversity Research Institute, Chinese Academy of Sciences Yezin Myanmar; 3 Forestry and Grassland Service Center, Pu’er Forestry Department, Pu’er, 665000, China Forestry and Grassland Service Center, Pu’er Forestry Department Pu’er China; 4 Institute of Forestry and Grassland Science of Pu’er, Pu’er, 665000, China Institute of Forestry and Grassland Science of Pu’er Pu’er China

**Keywords:** Agamid, Burma, cross-border, distribution, Draconinae, Pricklenape, taxonomy

## Abstract

Using an integrative dataset of both mitochondrial DNA and morphological data, we describe a new species of the genus *Acanthosaura* from the China-Myanmar border region in Menglian County, Pu’er, Yunnan Province, China. The new species, *Acanthosaura aurantiogularis***sp. nov**., is sister to *A. tongbiguanensis*, and it can be readily diagnosed from all recognized congeners by its bright orange gular coloration in the male, flesh colored oral cavity, moderate post-orbital spines and occipital spines, five or six well-developed nuchal crest scales, and a distinct dorsal coloration. We recommend further comprehensive phylogenetic studies of the genus and highlighted the understudied agamid diversity in northeastern Myanmar.

## Introduction

The agamid lizards of the genus *Acanthosaura* Gray, 1831, commonly known as Mountain Horned Dragons or Pricklenape Agamas, are widely distributed across Southeast Asia (Wood et al. 2010; Liu et al. 2020; Poyarkov et al. 2023). Recent taxonomic revisions have revealed a rich, undocumented diversity within the genus in China and nearby countries in northern Indochina (Ananjeva et al. 2025; Le et al. 2025; Ngo et al. 2025). In particular, several new species were described from the border regions in Yunnan Province, China and neighboring countries, including *A. brachypoda* Annanjeva, Orlov, Nguyen & Ryabov, 2011 from the China-Vietnam border in Honghe Prefecture of China and Lao Cai Province of Vietnam (Ananjeva et al. 2011; Liu et al. 2023), *A. tongbiguanensis* Liu & Rao, 2019 from the China-Myanmar border in Dehong Prefecture of China (Liu and Rao 2019), *A. rubrilabris* Liu, Rao, Hou, Orlov, Ananjeva & Zhang, 2022 from the China-Laos border region in Xishuangbanna of China and northern Laos (Liu et al. 2022; Poyarkov et al. 2023), and *A. longicauda* Liu, Rao, Hou, Orlov, Ananjeva & Zhang, 2022 from the Vietnam-Laos-China border in Pu’er of China and Dien Bien and Lai Chau provinces of Vietnam (Liu et al. 2023; Nguyen et al. 2025). Despite these continuous efforts, there are still regions in Yunnan Province that are not surveyed for the diversity of *Acanthosaura*, particularly those at the China-Myanmar border region in Pu’er.

During a herpetological survey of Pu’er in 2025, three specimens of the genus *Acanthosaura* were collected from Menglian County, Pu’er, Yunnan Province, very close to the China-Myanmar border. Both morphological comparisons and phylogenetic analyses confirm the distinctiveness of these specimens. Therefore, we describe them as a new species.

## Material and methods

### Sampling

**Figure 1.**
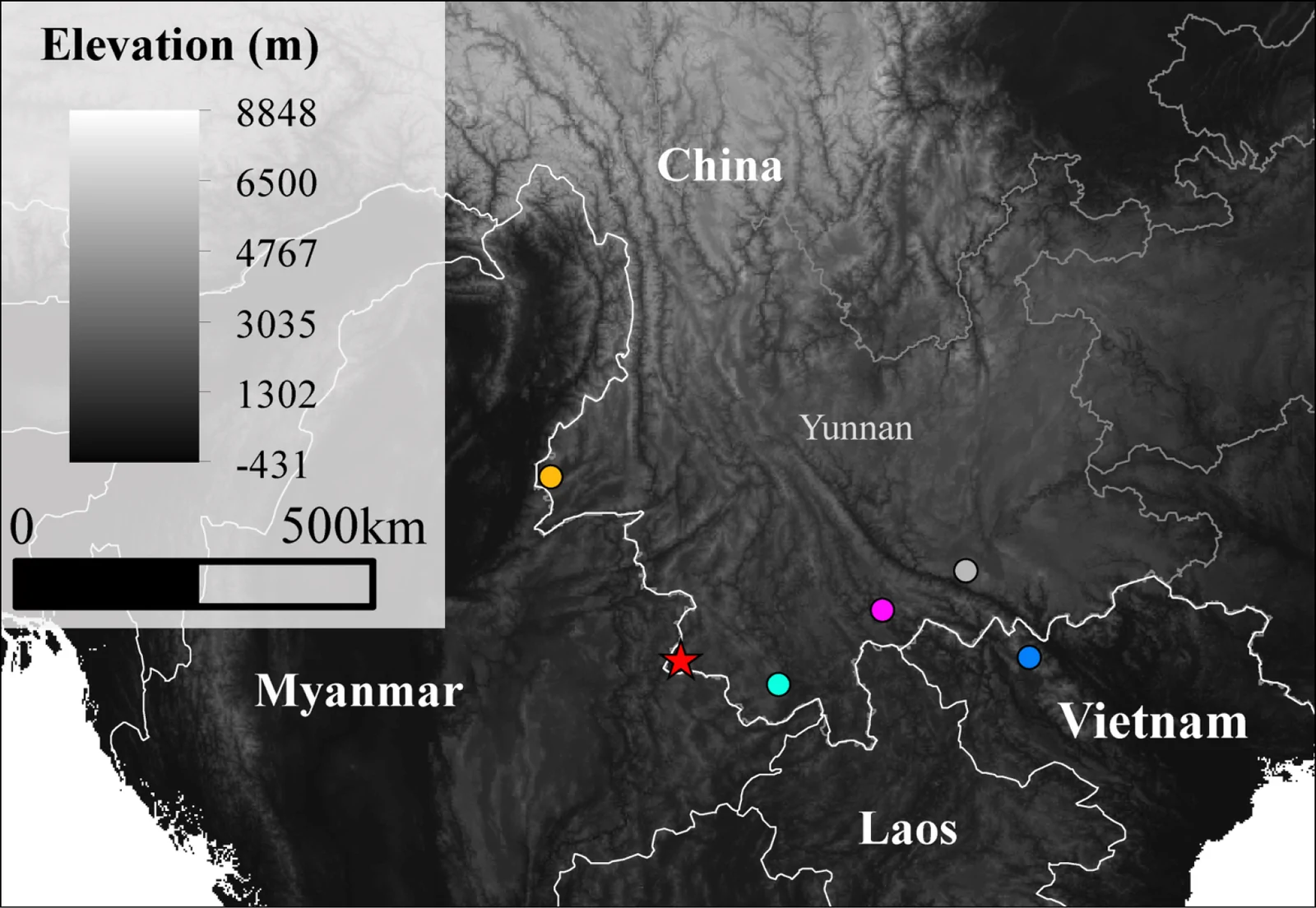
Type localities of species in the genus *Acanthosaura* in Yunnan Province of China and nearby countries in northern Indochina. Red star indicates the type locality of the new species, *Acanthosaura aurantiogularis* sp. nov. in Menglian County, Pu’er City, Yunnan Province, China. Circles represent type localities of recognized congeners, where different colors indicate different species, including *A. brachypoda* (dark blue), *A. liui* (gray), *A. longicauda* (violet), *A. rubeilabris* (turquoise), and *A. tongbiguanensis* (orange).

Three individuals of the genus *Acanthosaura* were collected from Menglian County, Pu’er, southwestern Yunnan Province, China (Fig. 1). Following euthanasia through overdose injection of MS222, liver tissues were taken from each individual and preserved in 95% ethanol, and all specimens were then fixed in 10% formalin solution and transferred to 75% ethanol after returning from the field. All newly collected specimens were deposited in the Kunming Natural History Museum of Zoology in Kunming Institute of Zoology, Chinese Academy of Sciences (**KIZ**). Additionally, voucher specimens of recognized congeners were examined in KIZ and California Academy of Sciences, USA (**CAS**). Morphological data of additional congeners were obtained from literature (Orlov et al. 2006; Wood et al. 2009, 2010; Ananjeva et al. 2011; Pauwels et al. 2015; Liu et al. 2020, 2023; Nguyen et al. 2018, 2019; Trivalairat et al. 2020, 2022).

### Morphological data

Morphometric data were measured using a digital caliper to the nearest 0.1 mm. The following morphometric characters were measured: snout–vent length (**SVL**), from tip of snout to the cloaca; tail length (**TAL**), measured from the tip of tail to the cloaca; head length (**HL**), from the tip of snout to the axis of jaw; head width (**HW**), measured as the widest width of head; head depth (**HD**), measured perpendicularly at the axis of jaw; internasal distance (**IN**), measured as the shortest distance between nares; snout–eye length (**SEL**), measured from the tip of snout to the anterior corner of eye; eye–nare distance (**END**), measured from the nare to the anterior corner of the eye; snout–limb distance (**SnL**), from the tip of snout to the base of forelimb at the insertion point when forelimb held at the right angle; tympanum diameter (**TD**), measured as the largest diameter; orbit diameter (**OD**), measured horizontally between the anterior-most and posterior-most edges of fine ciliary scales; interorbital distance (**IOD**), measured dorsally between the widest point; trunk length (**TRL**), measured between the axillary and the groin; brachium length (**BL**), measured between the forelimb insertion point at the axillary to the outermost point of the elbow when forearm held at the right angle; forearm and hand length (**FHL**), measured between the tip of finger IV and elbow when forearm held at the right angle; femur length (**FL**), measured from the hindlimb insertion point at the groin to the knee joint; and tibia and foot length (**TFL**), measured from the knee joint to the tip of toe IV when foot is straighten; tallest nuchal crest length (**TNCL**), measured between the tip and the posteroinferior end of the tallest nuchal crest; width of tallest nuchal crest at base (**WTNC**), measured at the base between the anterior and posterior edge of the tallest nuchal crest; length of post-orbital spine (**LPOS**), measured between the tip and the base of the post-orbital spine; length of occipital spine (**LOC**), measured between the tip and the base of the occipital spine; width of occipital spine at base (**WOS**), measured at the base between the anterior and posterior edge of the occipital spine; length of tallest dorsal crest (**LTDC**), measured between the tip and the posteroinferior end of the tallest dorsal crest; length of diastema (**LD**), measured between the posteroinferior edge of last enlarged nuchal crest and the anteroinferior edge of the first enlarged dorsal crest.

Pholidosis characters included: supralabials (**SL**), counted as the enlarged, modified labial scales of upper lip; infralabials (**IL**), counted as the enlarged, modified labial scales of lower lip; gular scales (**GU**), counted as the number of gular scales linearly from the first small gular scale posterior of mental (or chin shields if enclosed by chin shields) to the transverse gular fold (or equivalent position on neck); ventral scale counts (**VS**), counted as the number of ventral body scales linearly from the gular fold (or equivalent position of neck if gular fold is absent) to the cloaca along the medial line; number of small scale rows between nasal and first supralabial (**NSL**), counted as the fewest number of transverse scale rows between nasal and the first supralabial scale; middorsal crest scale count (**MC**), number of enlarged crest scales (both nuchal and dorsal) anterior of cloaca; number of enlarged nuchal crest scales (**NEN**), number of enlarged nuchal crests, anterior of the diastema; number of small scales of the diastema (**NSD**), number of fine scales between the posteroinferior edge of last enlarged nuchal crest scales and the anteroinferior edge of the first enlarged dorsal crest; subdigital lamellae under finger IV (**F4S**), counted as the subdigital lamellae between the tip of finger IV and the base of finger IV; subdigital lamellae under toe IV (**T4S**), counted as the subdigital lamellae between the tip of toe IV and the base of toe IV; and the development status of the dorsal crest (**DDC**). For analyses, Mann-Whitney U tests were performed using R (R core team 2021).

### Molecular analyses

Total genomic DNA was extracted from tissue samples stored in 95% ethanol. Tissue samples were digested using proteinaseK, and subsequently purified using standard phenol-chloroform protocols. Three mitochondrial genes (cytochrome *c* oxidase subunit 1[*COI*], cytochrome *b* [*Cytb*], and NADH dehydrogenase subunit 2 [*ND2*]) were amplified and sequenced using the published primers and PCR protocols (Che et al. 2012). PCR products were isolated through 1% agarose gel electrophoresis and further purified using Millipore Microcon Kits. Purified PCR products were then sent off for sequencing by the South China Barcoding Center. Outgroup selection followed a recent phylogenetic study of the family Agamidae (Wang et al. 2019; Scarpetta et al. 2025), which included *Diploderma* and *Calotes*. Homologous sequences of these outgroups and congeners of our putative new species were downloaded from GenBank (Table 1; Suppl. material 1).

**Table 1.** GenBank accession number used for the present study. Details on the voucher and locality of associated sequences are presented in Suppl. material 1. Newly generated data are in bold.

**Species**	**GenBank Accession No**.		
	***COI***	***Cytb***	***ND2***
*Diploderma luei*	HQ650554	KT920185	MK001433
*Calotes emma*	MT608024	–	DQ289460
*Acanthosaura crucigera*	–	–	MH777408
*A. crucigera*	–	–	MH777403
*A. crucigera*	–	–	MH777402
*A. crucigera*	MT607982	–	GU817389
*A. crucigera*	–	AY572887	HM143889
*A. crucigera*	–	AY572895	–
*A. armata*	AB266452	AB266452	AB266452
*A. aurantiacrista*	–	–	MH777406
*A. aurantiacrista*	–	–	MK798128
*A. aurantiacrista*	–	–	MK798129
*A. capra*	MK239022	PV658741	AF128498
*A. cardamomensis*	–	–	GU817397
*A. cardamomensis*	–	–	GU817400
*A. lepidogaster*	MK695195	AY572927	AF128499
*A. lepidogaster*	MK695194	AY572909	–
*A. lepidogaster*	OR102751	–	–
*A. lepidogaster*	OR102758	–	–
*A. lepidogaster*	OR102753	–	–
*A. hainanensis*	OR102772	KR092427	KR092427
*A. hainanensis*	OR102777	–	–
*A. brachypoda*	OR102766	–	–
*A. brachypoda*	OR102760	–	–
*A. brachypoda*	MK695182	–	–
*A. longicauda*	ON207531	–	–
*A. longicauda*	OR102782	–	–
*A. rubrilabris*	ON207532	–	–
*A. rubrilabris*	ON207542	–	–
*A. rubrilabris*	ON207545	–	–
*A. rubrilabris*	ON207547	–	–
*A. rubrilabris*	ON207548	–	–
*A. rubrilabris*	ON207549	–	–
*A. rubrilabris*	**PZ733441**	**PZ733434**	**PZ715330**
*A. rubrilabris*	**PZ733440**	**PZ733435**	**PZ715331**
*A. liui*	MT769763	–	–
*A. liui*	MT769764	–	–
*A. phongdienensis*	MK695205	–	–
*A. phongdienensis*	MK695207	–	–
*A. coronata*	MK695189	AY572896	–
*A. coronata*	MK695187	AY572897	–
*A. murphyi*	MK239026	–	–
*A. murphyi*	MK239025	–	–
*A. nataliae*	MK695202	AY572880	–
*A. nataliae*	MK695203	AY572881	–
*A. tongbiguanensis*	–	MN604012	–
*A. tongbiguanensis*	–	MN604013	–
*A. prasina*	–	MT376212	–
*A. prasina*	–	MT376212	–
*A. prasina*	–	AY572928	–
*A. cuongi*	PV646289	PV714672	–
*A. cuongi*	PV646288	PV658750	–
*A. grismeri*	PV646696	–	–
*A. grismeri*	PV646695	–	–
*A. meridiona*	–	–	MH777404
*A. meridiona*	–	–	MH777405
*A. meridiona*	–	–	MH777407
*A. aurantiogularis* sp. nov.	**PZ733437**	–	**PZ715332**
*A. aurantiogularis* sp. nov.	**PZ733439**	**PZ733436**	**PZ715333**
*A. aurantiogularis* sp. nov.	**PZ733438**	–	**PZ715334**

Phylogenetic analysis was inferred based on concatenated alignment of all three mitochondrial genes, using both maximum likelihood (ML) and Bayesian inference (BI) methods. All genes were aligned using MEGA 11 (Tamura et al. 2021). Maximum likelihood analyses were conducted on the IQ-TREE online server (Trifinopoulos et al. 2016), with auto-selected substitution models, which were TIM+F+I+G4 for *COI*, TN+F+G4 for *ND2*, and K3Pu+F+G4 for *Cytb*, and 10,000 iterations of Ultrafast bootstrap and SH-aLRT branch tests. Bayesian inference was conducted using MrBayes (v. 3.2.7a) on CIPRES (Miller et al. 2010), with best substitution model selected using ModelFinder (Kalyaanamoorthy et al. 2017). HKY+gamma was selected for *Cytb* and *ND2*, and GTR+I was selected for *COI.* Two independent Markov Chain Monte Carlo analyses were run, each with four Metropolis-coupled chains, a melting temperature of 0.02, and an exponential distribution with a rate parameter of 25 as the prior on branch lengths. Each run was conducted with 10 million generations and sampled every 1000 generations, discarding the first 25% of trees as burn-in. Convergence of runs was confirmed with TRACER v. 1.7.0 (visual convergence and effective sampling size > 200; Rambaut et al. 2018). The uncorrected genetic distance among individuals was calculated using MEGA 11 with the pairwise deletion option (Tamura et al. 2021) for each of the three mitochondrial genes used.

## Results

### Molecular results

**Figure 2.**
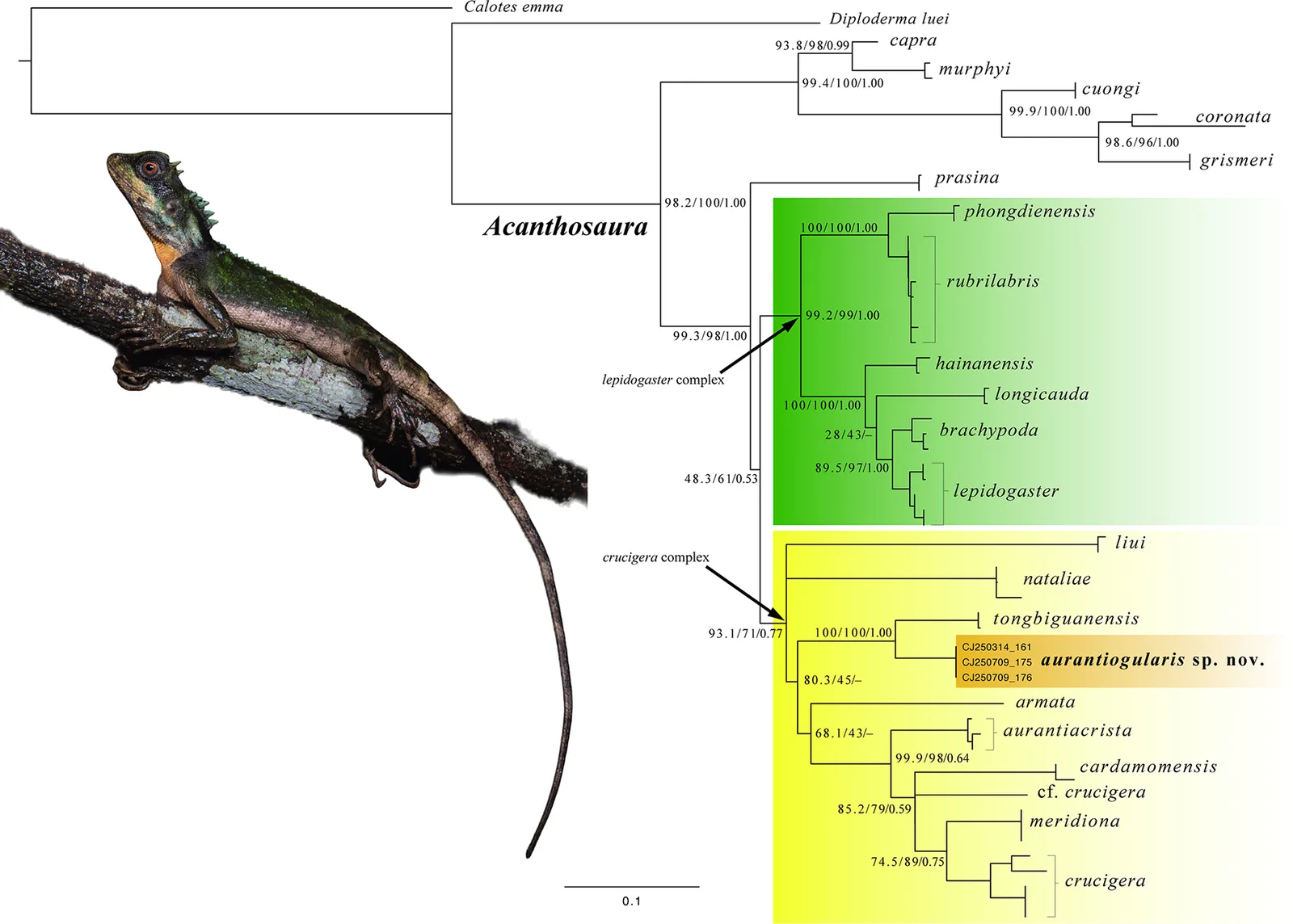
Mitochondrial genealogy of the genus *Acanthosaura* inferred from a concatenated alignment of *COI*, *Cytb*, and *ND2* genes. Nodal supports are given as Ultrafast bootstrap/ SH-aLRT branch tests/Bayesian posterior probability, respectively, and the Bayesian posterior probabilities were mapped onto the topology from IQ-TREE analyses. Nodal supports that unify individual species were all strongly supported and omitted, and the intraspecific relationships are also omitted from the phylogeny. “–” indicates not-applicable Bayesian supports due to differential topology between Bayesian and IQ-TREEanalyses. The two involved species complexes of the genus (i.e., lepidogaster complex and crucigera complex) are highlighted by green and yellow coloration, respectively. The inserted photo is of the holotype of *Acanthosaura aurantiogularis* sp. nov. in life, which was photographed by ZYL.

*Acanthosaura capra* Günther, 1861, *A. murphyi* Nguyen, Do, Hoang, Nguyen, McCormack, Nguyen, Orlov, Nguyen & Nguyen, 2018, *A. cuongi* Ngo, Le, Nguyen, Nguyen, Nguyen, Phan, Nguyen, Ziegler & Do, 2025, *A. coronata* Günther, 1861, and *A. grismeri* Le, Nguyen, Nguyen, Ziegler, Do & Ngo, 2025 form a strongly supported clade (nodal supports 99.4/100/1.00, which are Ultrafast bootstrap/SH-aLRT test/Bayesian posterior probability, abbreviated herein; Fig. 2). Within this clade, *A. capra* and *A. murphyi* constitute a subclade, followed by *A. cuongi* (99.9/100/1.00) and eventually the subclade unifying *A. grismeri* and *A. coronata* (98.6/96/1.00).

**Figure 3.**
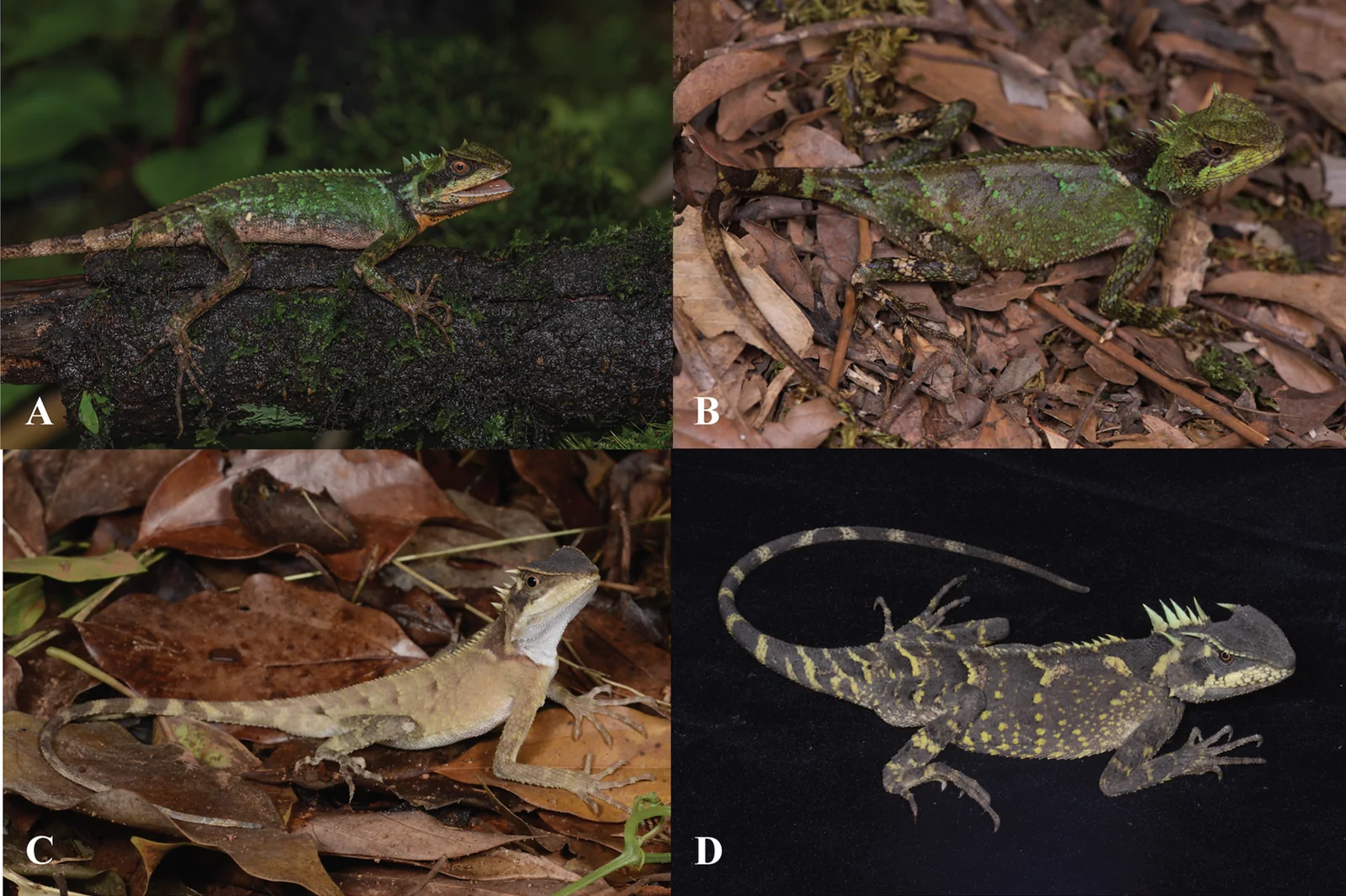
Comparison between males (left column) and females (right column) of *Acanthosaura aurantiogularis* sp. nov. (top row, **A**, **B**) and closely related *A. tongbiguanensis* (bottom row, **C**, **D**) in life. Photos by ZYL and KW.

While the remaining sampled species form a clade (99.3/98/1.00), relationships among them remain largely unresolved. Of these species, the *A. lepidogaster* (Cuvier, 1829) complex is recovered as monophyletic with strong supports (99.2/98/1.00), which contains *A. brachypoda*, *A. hainanensis* Boulenger, 1900, *A. lepidogaster*, *A. longicauda*, *A. phongdienensis* Nguyen, Jin, Vo, Nguyen, Zhou, Che, Murphy & Zhang, 2019, and *A. rubrilabris* (Fig. 3). *Acanthosaura crucigera* Boulenger, 1885 complex is recovered as monophyletic with moderate support (93.1/71/0.77). Specimens of the putative new species from Menglian County form a monophyletic group with strong support (100/100/1.00), and they are sister to *A. tongbiguanensis* with strong support (100/100/1.00). Relationships of the remaining species of the *A. crucigera* complex remain largely unresolved.

The uncorrected genetic distances among specimens of the putative new species are minimal (< 1%), but they show substantial divergence from its closest relative *A. tongbiguanensis* (8.2–8.3% in *Cytb*) and all other sampled congeners (> 14% in *Cytb*, *COI*, and *ND2*; Suppl. material 2).

### Morphological results

Morphologically, the newly collected specimens of the putative new species from Menglian County align with the *A. crucigera* complex, including having moderate length of post-orbital and occipital spines, a transverse gular fold, and well-developed nuchal and dorsal crests. Additionally, the male specimen of the putative new species from Menglian County share similar gular coloration with *A. rubrilabris*. However, it can be diagnosed from the above-mentioned species and all remaining congeners by a combination of morphological characters, including a distinct gular coloration, number of nuchal crest scales, relative length of post-orbital and occipital spines, and a distinct dorsal coloration (Table 2).

**Table 2.** Morphological comparison between the new species *Acanthosaura aurantiogularis* sp. nov. and closely related or morphologically similar congeners. Comparison data or congeners were obtained from literature (Liu et al. 2023) and examination of specimens (i.e., *A. crucigera* and *A. liui*).

**Species**	***A. aurantiogularis* sp. nov**.	***A. tongbiguanensis***	***A. crucigera***	***A. rubrilabris***	***A. lepidogaster***	***A. liui***	***A. longicauda***
Relative length of post-orbital spine (LPOS/HL)	11.0–15.1%	11.2–14.9%	7.0–8.0%	5.0–12.0%	6.0–13.0%	6.5–9.7%	8.0–12.0%
Development status of dorsal crest (DDC)	Well-developed	Well-developed	Well-developed	Feeble	Feeble	Well-developed	Feeble
Number of enlarged nuchal crests (NEN)	5 or 6	6–8	9 or 10	3–6	6–9	7 or 8	6 or 7
Number of middorsal crest scale (MC)	41	45–52	50–52	49 or 50	50–54	34–42	47–52
Number of lamellae under toe IV (T4S)	23 or 24	25–28	21–25	18–25	21–25	22–26	22–25
Connectivity between eye stripe and nuchal collar pattern	Disconnected	Connected	Connected	Connected	Connected	Connected	Connected
Coloration of dorsum	Green	Brown	Brown	Green	Green	Gray	Green
Coloration of oral cavity	Flesh color	Flesh color	Flesh color	Bluish gray to purplish black	Bluish gray to purplish black	Yellow or flesh color	Bluish gray to purplish black

### Taxonomic account

#### Acanthosaura aurantiogularis

Taxon classificationAnimaliaSquamataAgamidae

Wang, Lu & Che sp. nov.

C6C9157C-9982-571E-9B70-B052641957EA

https://zoobank.org/C3A96BC6-DE01-468F-BE1A-2E04C90CB2F3

**Figure 4.**
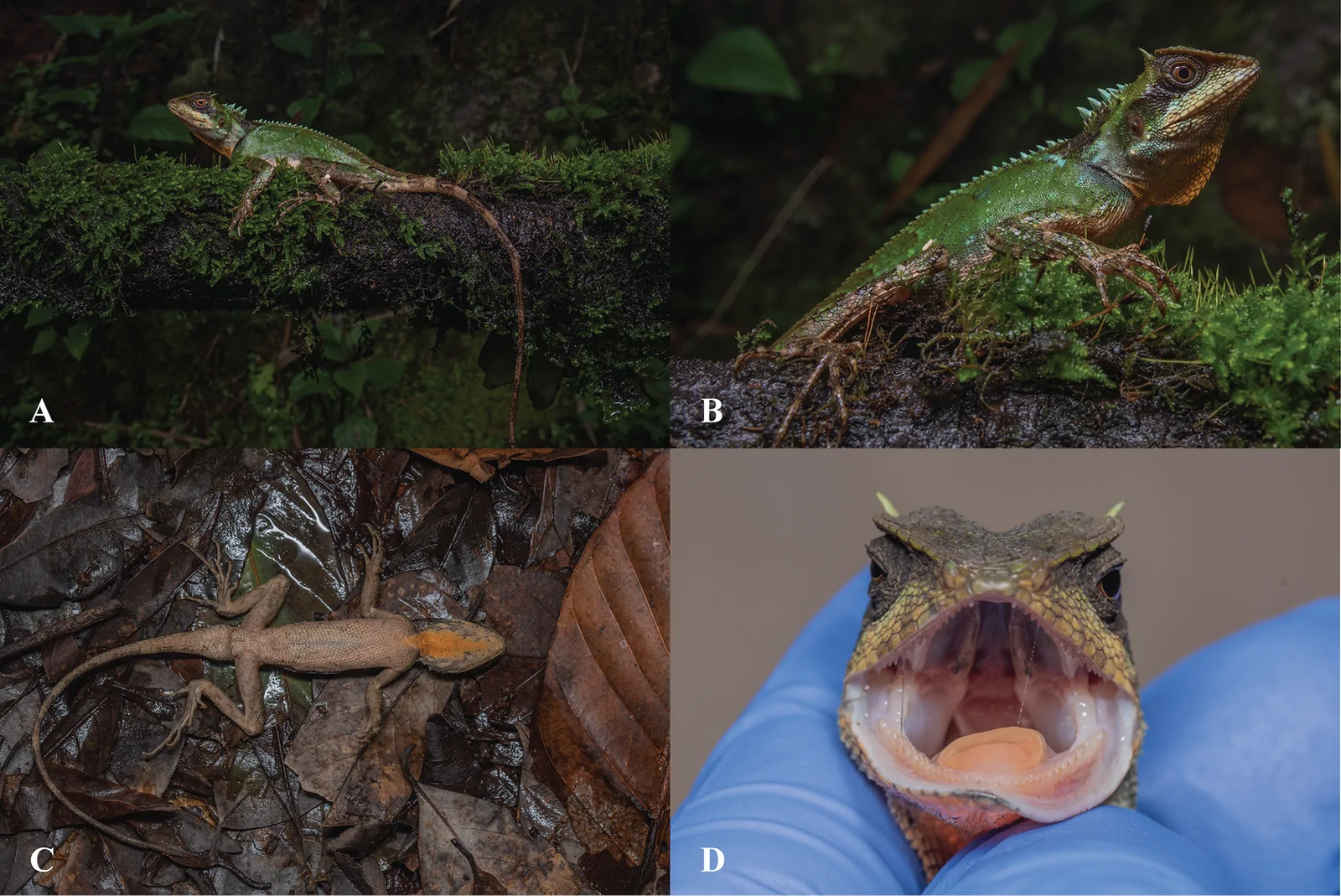
Holotype male of *Acanthosaura aurantiogularis* sp. nov. (KIZ 61664) in life, showing lateral overview (**A**), lateral closeup of body and head (**B**), ventral overview (**C**), and closeup of oral cavity (**D**). Photos by ZYL.

**Figure 5.**
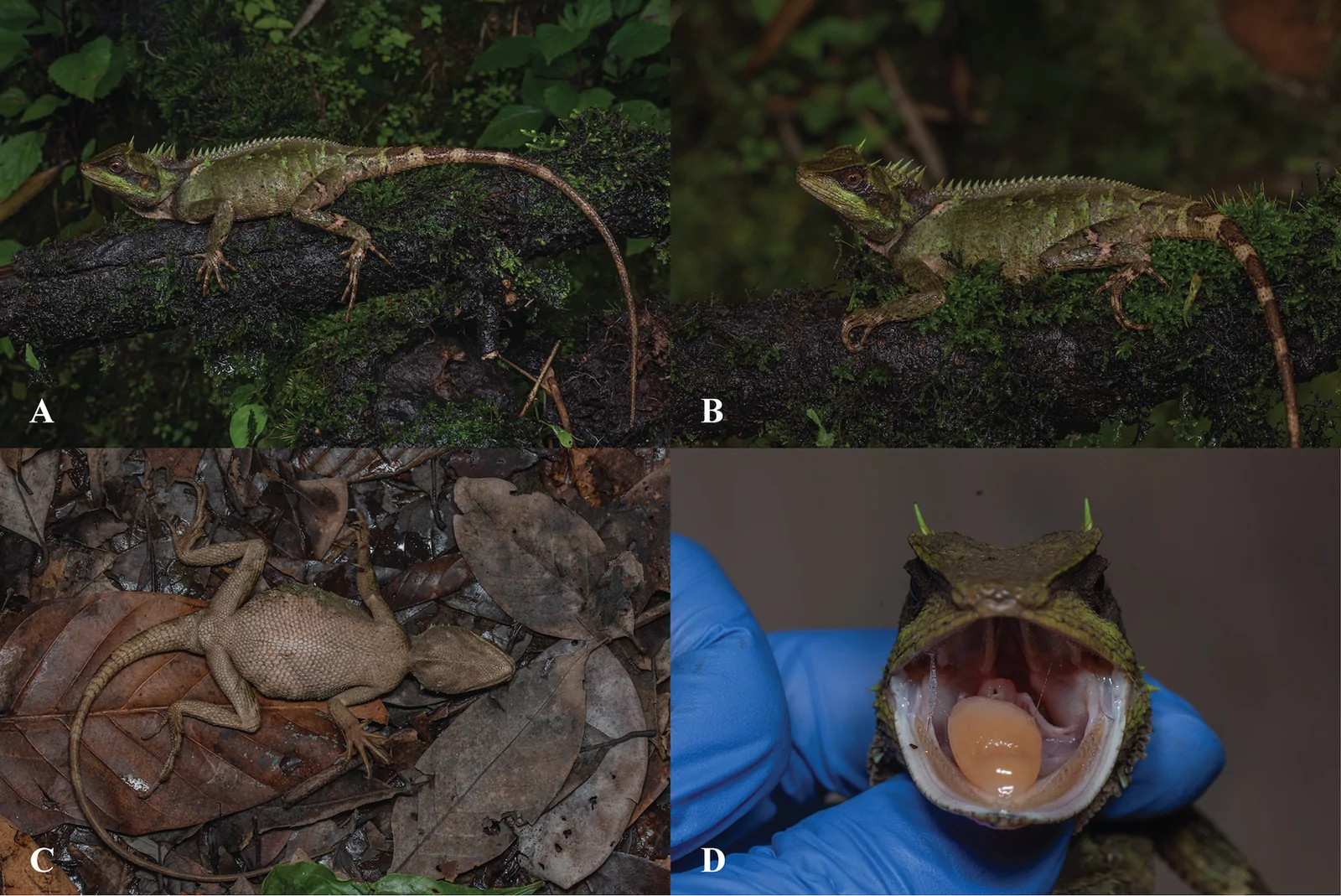
Paratype female of *Acanthosaura aurantiogularis* sp. nov. (KIZ 61665) in life, showing lateral overview (**A**), lateral closeup of body and head (**B**), ventral overview (**C**), and closeup of oral cavity (**D**). Photos by ZYL.

**Figure 6.**
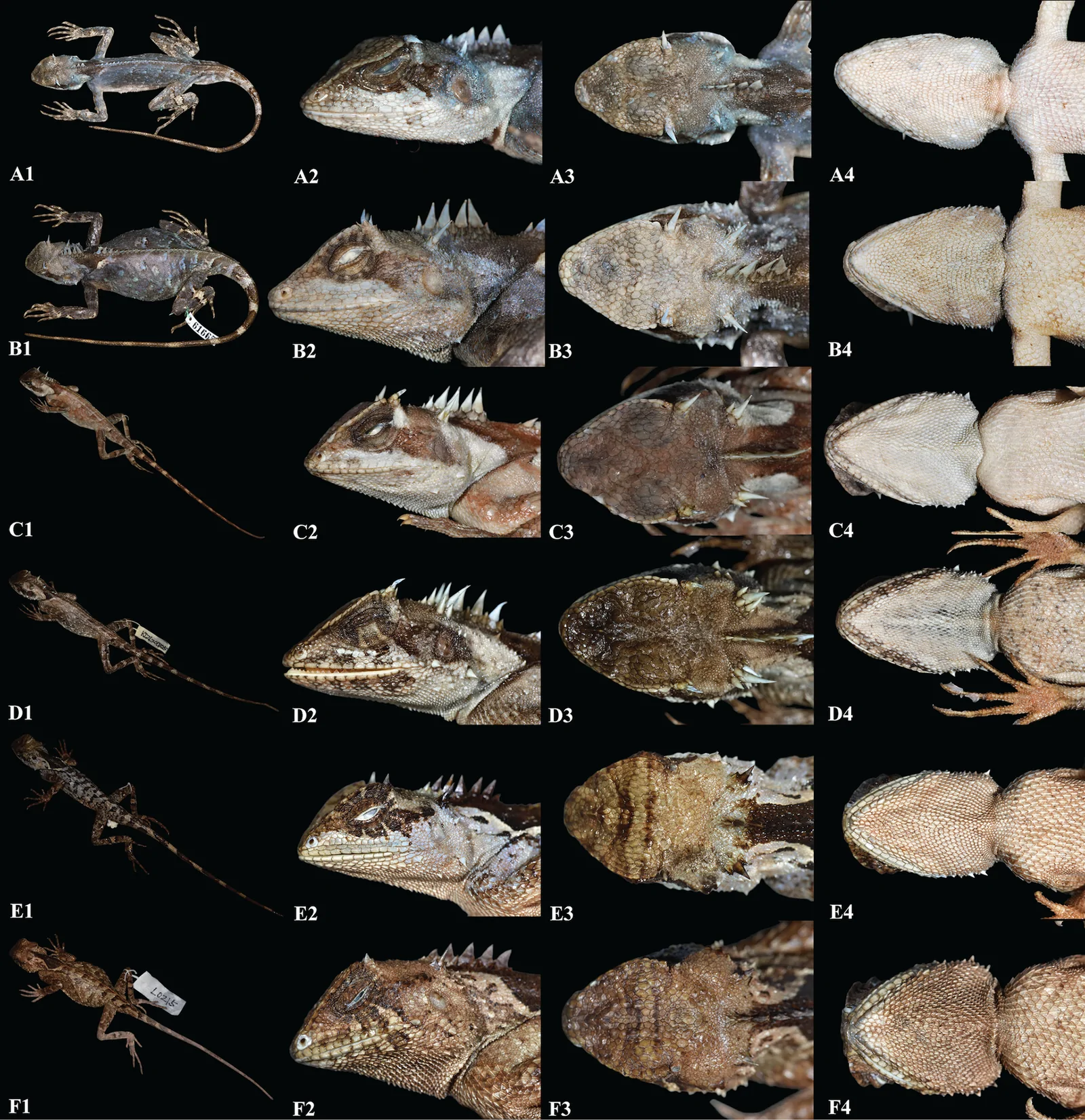
Morphological comparisons of males (**A, C**, **D**) and females (**B, D**, **E**) among types of *Acanthosaura aurantiogularis* sp. nov. (**A**. KIZ 61664; **B**. KIZ 61665) and types of closely related/closely distributed species, including *A. tongbiguanensis* (**C**. KIZ L201804; **D**. KIZ L201805) and *A. rubrilabris* (**E**. KIZ L0214; **F**. KIZ 0215), showing the dorsolateral overview (1), closeup of lateral head (2), closeup of dorsal head (3), and closeup of ventral head (4). Photos by KW.

**Figure 7.**
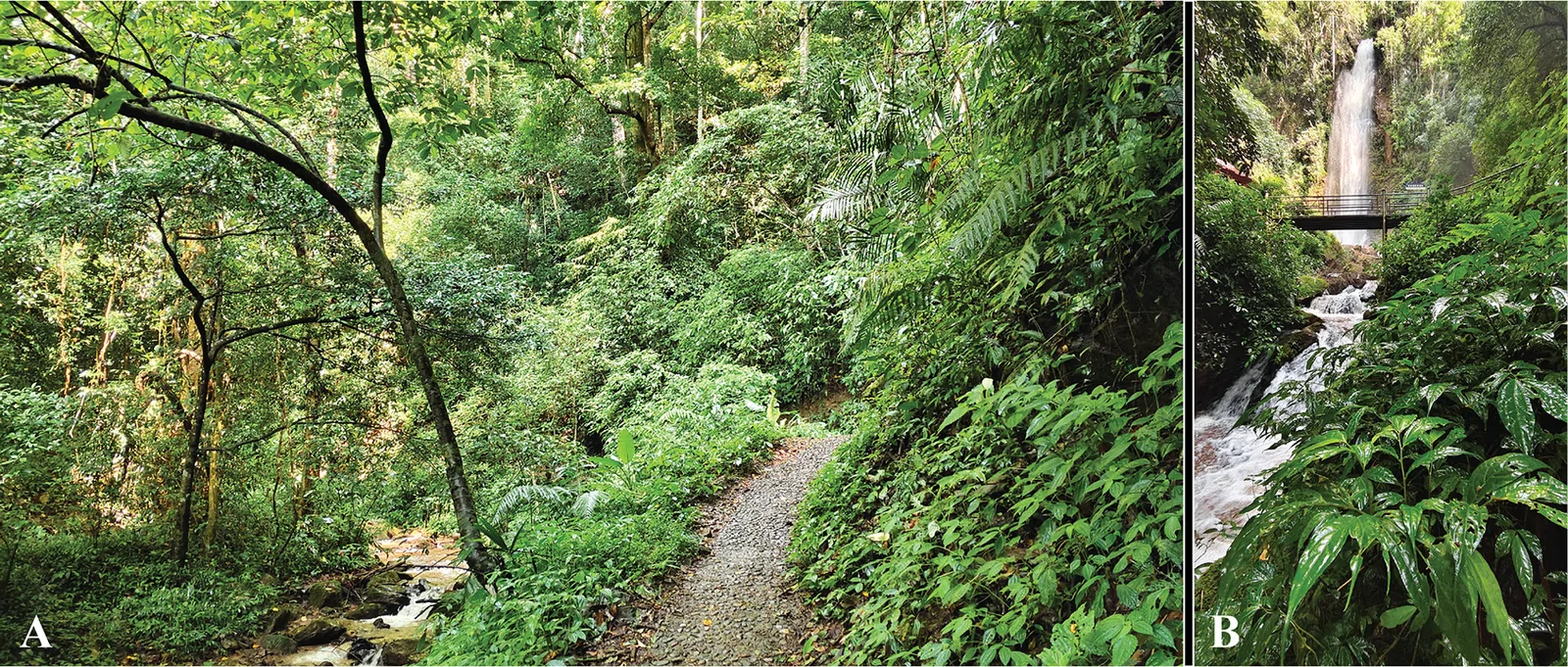
Macro- (**A**) and micro-habitat (**B**) of the new species near the Mengma Waterfall in Menglian County, Pu’er City, Yunnan Province, China. Photos by ZYL.

Table 3; Figs 4, 5, 6, 7

##### Material examined.

***Holotype***. China • ♂; Yunnan Province, Pu’er, Menglian County, Fuyan Township, Yinggou Village; 22.44792328°N, 99.44631053°E, elevation 1154 m (GCJ02); 23 July 2025; Zhuo-Yu Lu, Xian-Kun Huang, Xiang-Jin Liu, and Ming-Ru Su leg.; GenBank: PZ733437 and PZ715332; KIZ 61664. ***Paratypes***. China • 1 ♀; Yunnan Province, Pu’er, Menglian County, Mengma township, Mengma Waterfall; 22.21642307°N, 99.38063466°E, elevation 1061 m (GCJ02); 20 July 2025; Zhuo-Yu Lu, Xian-Kun Huang, Xiang-Jin Liu, and Ming-Ru Su leg.; GenBank: PZ733438 and PZ715334; KIZ 55233; • 1 ♀; same collection data as the proceeding, GenBank PZ733439, PZ733436, PZ715333; KIZ 61665.

**Table 3.** Morphometric and pholidosis data of the type series of the new species *Acanthosaura aurantiogularis* sp. nov. Detailed abbreviations of morphological characters used are the following: snout–vent length (SVL), tail length (TAL), head length (HL), head width (HW), head depth (HD), internasal distance (IN), snout–eye length (SEL), eye–nare distance (END), snout–limb distance (SnL), tympanum diameter (TD), orbit diameter (OD), interorbital distance (IOD), trunk length (TRL), brachium length (BL), forearm and hand length (FHL), femur length (FL), tibia and foot length (TFL), tallest nuchal crest length (TNCL), length of post-orbital spine (LPOS), length of occipital spine (LOC), length of the longest dorsal crest (LLDC), length of diastema (LD); supralabials (SL), infralabials (IL), gular scales (GU), ventral scale counts (VS), number of small scale rows between nasal and first supralabial (NSL), middorsal crest scale count (MC), number of enlarged nuchal crests (NEN), number of small scales of the diastema (NSD), subdigital lamellae under finger IV (F4S), subdigital lamellae under toe IV (T4S), and development status of dorsal crests (DDC).

**Voucher number**	**KIZ 61664**	**KIZ 61665**	**KIZ 55233**
Type status	Holotype	Paratype	Paratype
Sex	M	F	F
SVL	80.0	116.8	107.5
TAL	–	195.0	–
HL	23.5	35.8	31.2
HW	15.9	24.5	20.6
HD2	12.5	19.5	15.7
SnL	32.7	43.8	40.7
SEL	9.2	13.9	12.3
END	5.7	9.2	7.9
IN	5.1	8.0	6.7
OD	6.6	7.3	7.7
TD	3.8	5.2	4.9
IOD	13.9	19.4	17.1
BL	15.3	22.0	21.5
FHL	31.9	43.9	41.4
F4CL	2.2	4.0	3.8
T4CL	2.4	2.6	2.7
FLLS	44.4	60.5	58.8
FLL	47.2	65.9	62.9
FL	23.5	31.9	28.7
TFL	50.3	67.8	66.5
F4L	10.7	14.5	13.6
T4L	15.7	22.2	22.1
HLL	70.7	97.8	91.6
TRL	36.8	62.7	54.3
TNCL	3.5	6.7	4.3
WTNC	2.0	2.6	2.5
LLOC	2.2	5.7	3.9
WOS	1.0	1.4	1.3
LOPS	2.6	5.4	4.4
LTDC	1.7	5.6	3.0
LD	4.1	5.8	6.4
SL	10/10	11/11	10/10
IL	10/12	11/12	11/11
NSL	1/1	1/1	0/0
GU	37	32	34
VS	63	56	58
MC	41	41	41
F4S	19	19	20
T4S	23	24	23
NEN	6	5	5
DDC	Well-developed	Well-developed	Well-developed

##### Etymology.

The specific epithet *aurantiogularis* is a compound Neo-Latin adjective derived from the Latin *aurantius*, meaning “orange-colored,” and *gularis*, meaning “the throat”. This nomenclature is a direct descriptor of the lizard’s most striking and diagnostic morphological feature: the vibrant, unmarked orange-yellow gular pouch in males. The suggested Chinese common name is “橙喉棘蜥” (Pinyin: Chéng Hóu Jí Xī), and the suggested English common name is Orange-throated Pricklenape.

##### Diagnosis.

*Acanthosaura aurantiogularis* sp. nov. can be diagnosed from congeners by a combination of the following morphological characters: (1) tail short, TAL 167% SVL in female; (2) length of post-orbital and occipital spines moderate, LOPS 11.0–15.1% HL, LLOC 9.5–15.9% HL; (3) nuchal crest erected, 5 or 6, discontinuous from dorsal crests, TNCL 13.8–18.7% HL; (4) diastema short, composed of 6–8 small scales; (5) dorsal crest erected, anterior most one shorter than nuchal, decreasing in length posteriorly; (6) enlarged middorsal crest scales 41; (7) gular pouch moderately developed, transverse gular fold present; (8) dorsal body scales heterogeneous, enlarged, conical scales scattered on dorsum, some clustering into short vertical or oblique rows; (9) dorsal background olive green, with enlarged dorsal scales bright green; (10) black eye patch discontinuous from black collar pattern on neck; (11) lips bright green or whitish green; (12) ventral body distinct from dorsal in coloration, dark grayish white in females, off-white in male; (13) large, elongated gular spot present in male only, bright orange, with irregular, cloud-like, dark gray patterns on lateral gular; and (14) oral cavity and tongue pink-flesh colored.

##### Comparison.

*Acanthosaura aurantiogularis* sp. nov. is most similar to *A. tongbiguanensis* phylogenetically based on available data, and both species are geographically close in distribution. However, *A. aurantiogularis* sp. nov. can be diagnosed from the latter by having fewer lamellae under IV toe (23 or 24 vs. 25–28, Mann-Whitney U test p < 0.05), fewer enlarged crest scales (MC 41 vs. 45–52, Mann-Whitney U test p < 0.05), a distinct dorsal background coloration (green vs. light brown or blackish brown), the dark eye patch and the neck collar pattern discontinuous (vs. continuous), and by the presence of orange gular spot in males (vs. absence).

Phylogenetically, *A. aurantiogularis* sp. nov. belongs in the *A. crucigera* complex, but it can be readily diagnosed from *A. crucigera* (and its synonym, *A. horrescens* Lönnberg, 1916) by having longer post-orbital spines (LOPS 11.0–15.1% HL vs. 7.0–8.0%), fewer nuchal crests anterior of the diastema (5 or 6 vs. 9 or 10), having a distinct gular spot in males (vs. absent), a distinct coloration around eyes (a distinct blackish patch expands across eye vs. thin radial stripes around eyes), and a distinct dorsal background coloration (green vs. brown); from *A. aurantiacrista* Trivalairat, Kunya, Chanhome, Sumontha, Vasaruchapong, Chomngam & Chiangkul, 2020, *A. bintangensis* Wood, Grismer, Grismer, Ahmad, Onn & Bauer, 2009, *A. meridiona* Trivalairat, Sumontha, Kunya & Chiangkul, 2022, *A. phuketensis* Pauwels, Sumontha, Kunya, Nitikul, Samphanthamit, Wood & Grismer, 2015, and *A. titiwangsaensis* Wood, Grismer, Grismer, Ahmad, Onn & Bauer, 2009 by the presence of distinct gular coloration (orange vs. yellow, white, or black), fewer nuchal crest scales anterior of the diastema (5 or 6 vs. 8–10), and a distinct dorsal coloration (green vs. brown).

Morphologically, the new species resembles *A. rubrilabris*, both of which have an overall green coloration and an orange gular region in males. Furthermore, both species are found in Pu’er, Yunnan Province. However, *A. aurantiogularis* sp. nov. can be readily diagnosed from *A. rubrilabris* by having longer post-orbital spines (LOPS 15.1% HL in males, 11.0–14.0% in females vs. < 12% in males, < 9% in females), much better developed dorsal crests (vs. underdeveloped and feeble), and a distinct oral coloration in both sexes (flesh color vs. bluish gray to purplish black).

For other species that are closely distributed, including *A. lepidogaster*, *A. liui* Liu, Hou, Mo & Rao, 2020, and *A. longicauda*, the new species differs from all by the presence of a distinct orange gular spot in males (vs. absence). Additionally, *A. aurantiogularis* sp. nov. differs from *A. longicauda* and *A. lepidogaster* by the presence of a transverse gular fold in life (vs. absence) and a distinct oral coloration in life (pink-flesh color vs. purplish black); and from *A. liui* by having a longer post-orbital spines (LOPS 11.0–15.1% HL vs. < 10.7%), a distinct dorsal head coloration (green vs. grayish white), and a disconnection between the brownish black eye stripe and the nuchal collar (vs. connected).

##### Description of holotype.

Young adult male, SVL 80.0 mm, body not compressed, cross-section near triangular; tail slender, bulgy posteriorly at base, incomplete at tip; limbs relatively long, forelimb length 56.4% SVL, HLL 92.2% SVL.

Head robust, HW 67.6% HL, HD 53.5% HL, HD 79.1% HW, SEL 39.0% HL. Rostral rectangular, length three times longer than height, bordering five small scales excluding supralabials; supralabials smooth, 10/10, sixth to ninth inferior of eye; dorsal head scales heterogeneous, all keeled; keels on medial snout forming a weak lateral ridge anterior of eye position; nasal oval, single scale row away from rostral and first supralabial; loreal nearly homogeneous, keeled, seven linearly between nasal and anterior-most edge of ciliaries; canthus rostralis distinct, 11 scales along canthus ridge between nasal and post-orbital spine, slightly overlapping; suborbital scale rows three, medial row slightly smaller, all keeled; eye surrounded by fine ciliaries; post-orbital spine distinct, LOPS 11.0% HL, shorter than occipital spine, LPOS 51.8% LOC; tympanum oval, longer diameter vertically, exposed, TD 57.1% OD; distinctively enlarged, conical scales post rictus, 6/9; occipital spine surrounded by four smaller conical scales at base; no supratympanic spines. Nuchal crest six, all erected, laterally compressed, triangular shaped, increasing size posteriorly, TNCL 14.7% HL, supported by distinctively keeled, smaller scales inferiorly on both sides, separated from dorsal crest by distinct diastema; diastema short, LD 17.6% HL, comprising eight small scales; dorsal crest triangular shaped, laterally compressed, anterior dorsal crest scales about same size as second nuchal, gradually decreasing in size posteriorly, all supported by distinctively keeled scales inferiorly on both sides. Shoulder fold present, shallow; dorsal background scales of body fine, all moderately keeled, nearly homogeneous, intermixed with enlarged, conical scales; some enlarged conical scales in clusters or oblique rows near the dorsal crest. Forelimbs scales nearly homogeneous, all distinctively keeled, with keels of individual scales aligned forming continuous keels from proximal to distal end; scales of dorsal thigh somewhat heterogeneous, keels of individual scales less organized; scales of crust same as forelimbs. Dorsal tail scales moderately keeled anteriorly, strongly keeled posteriorly.

Mental scale small, not enclosed by first pair of chin shields; infralabials smooth, smaller than supralabials, 10/12; chin shields relatively small, smooth, 5/5; remaining gulars nearly homogeneous, slightly enlarged toward medial-central region, all distinctively keeled, intermixed with a few enlarged subconical scales posterolaterally. Transverse gular fold feeble, gular pouch weak. Ventral body scales nearly homogeneous, larger than background dorsals, all distinctively keeled. Ventral limb scales weakly keeled; ventral tail scales all distinctively keeled, with keels aligned into lateral ridges.

##### Coloration.

Dorsal surfaces of the head, limbs, and body are dark green. A distinct blackish brown eye patch extents from the anterior loreal across the eye to the anterior surface of tympanum. Lips are off-white, distinct from the background green color and blackish eye stripe. The lateral surface of the neck is light whiteish green. A distinct blackish brown collar patch is present on the diastema, extending posteriorly across the dorsal neck and inferiorly along the shoulder fold to clavicle region. The color of enlarged conical scales of dorsum is lighter than background scales, some of which are even bluish green. Faint brown, oblique streaks are present along dorsum, clearer posteriorly. Dorsal coloration gradually transitions into light brown posterior of cloaca. The tail is banded with dark brown, with the bands gradually fading posteriorly. The dorsal surface of limbs is patterned with dirty brown, irregularly shaped transverse streaks or patches.

The background color of the ventral surfaces of the head, body, limbs, and tail is flesh color. A distinct, spear-like, orange colored gular spot is present on the ventral head, with its tip starting at about fifth infralabial and the end extending on anterior chest. Faint, marble shaped dark gray patterns are present abutting the orange gular spot laterally. The oral cavity and tongue are both flesh color.

##### Variation.

Morphometric and pholidosis variation is summarized in Table 3. Additionally, sexual dichromatism is evident despite the small sample size. Specifically, females lack the orange gular spot, the lips are bright green instead of white, and the ventral coloration is white.

##### Natural history.

All specimens were collected at night , and the paratypes were found by a medium sized stream (about 2 m in width) downstream from a waterfall, sleeping on stems of plants about 1 m above the ground (Fig. 7). The waterfall was a tourist site, but it was still surrounded by well-preserved evergreen broadleaf forest. Agamids of other genera (i.e., *Calotes emma* and *Paracalotes microlepis*) were collected not too far away from the micro-habitat of the new species. Currently, the new species is known from its type locality, but given the close geographic distance and continuous habitats, it is expected across the border in northern Shan State, Myanmar.

## Discussion

With the discovery of our new species, seven species of *Acanthosaura* are recorded from China, including *A. aurantiogularis*, *A. brachypoda*, *A. hainanensis*, *A. lepidogaster*, *A. liui*, *A. longicauda*, and *A. tongbiguanensis* (Liu and Rao 2019; Liu et al. 2020, 2022, 2023). Taxonomic works on the genus *Acanthosaura* have relied on morphological data for a long time (Boulenger 1885; Smith 1935; Taylor 1963; Wood et al. 2009, 2010; Ananjeva et al. 2011; Pauwels et al. 2015), and it was not until more recently taxonomic studies started to integrate molecular data (Liu and Rao 2019; Ananjeva et al. 2020; Liu et al. 2020, 2022, 2023; Trivalairat et al. 2020, 2022; Ngo et al. 2025). However, unlike other species-rich genera of the same subfamily (McGuire and Heang 2001; Solovyeva et al. 2014; Wang et al. 2019), there is no comprehensive molecular study on the phylogenetic relationship of the genus to date. This is partly due to the sporadic, non-overlapping genes used for the past taxonomic studies (Table 1) as well as the difficulty in acquiring comprehensive sampling cross multiple nations. Our study is the first attempt to integrate available mitochondrial data of the genus into a single phylogenetic analysis. To date, some species of the genus still lack molecular data (i.e., *A. bintangensis*, *A. phuketensis*, and *A. titiwangsaensis*; Ananjeva et al. 2025; Uetz et al. 2026), and relationships among species, especially within the *A. crucigera* complex, remain unresolved (Fig. 2). Future collaborative studies are needed to fill the gap on molecular data and reconstruct and resolve the phylogeny of the genus *Acanthosaura*.

Along with the continuous discoveries of other new agamid species from the China-Myanmar border region (Liu and Rao 2019; Liu et al. 2026; Xu et al. 2024), our results further suggest a unique yet understudied diversity of agamid lizards from Myanmar. The ongoing military conflicts between the central government of Myanmar and the Self-Administrated Zones close to China border greatly hinders biodiversity research and conservation. We speculate that the herpetofauna diversity of Myanmar is much higher than currently recognized, and many of China’s “endemic” species reported from the Chinese side of the border likely also occur in adjacent Myanmar as well (i.e., *Acanthosaura tongbiguensis*, *Cyrtodactylus dianxiensis*, and *Alcalus tongbiguanensis*), as supported by the recent studies of other groups (i.e., *Ptyctolaemus tongbiguanensis*, Deepak 2026). With the rapid deforestation in Myanmar, particularly regions close to the Chinese border, future surveys are urgently needed to document the biota in Myanmar and formulate conservation actions accordingly.

Currently, the genus *Acanthosaura* still suffers from the lack of robust morphological analyses, and the available study is limited to *A. lepidogaster* complex only (Liu et al. 2023). The limited sample size of our new species and other congeners (such as *A. liui* and *A. tongbiguanensis*) prevent us from conducting morphometric analyses of the group. Future continuous surveys are needed to obtain a better voucher representation for this elusive agamid group, and comprehensive morphological study using robust statistical analyses (Chan and Grismer 2021) are needed.

## Supplementary Material

XML Treatment for Acanthosaura aurantiogularis

## Additional information

### Artificial Intelligence (AI) use

The authors accept full responsibility for the content of the manuscript, including the disclosure of any use of AI.

No AI tools were used in the preparation of this manuscript.

### Author ORCIDs

K. Wang https://orcid.org/0000-0002-6736-3346

J. Che https://orcid.org/0000-0003-4246-6514

### Data availability

All of the data that support the findings of this study are available in the main text or Supplementary Information.

## Supplementary materials

Supplementary material 1 Voucher and locality information Data type xlsx Explanation note Detailed voucher and locality information associated with the Genbank accession numbers used. https://binary.pensoft.net/file/1756256This dataset is made available under the Open Database License (http://opendatacommons.org/licenses/odbl/1.0/). The Open Database License (ODbL) is a license agreement intended to allow users to freely share, modify, and use this Dataset while maintaining this same freedom for others, provided that the original source and author(s) are credited. Kai Wang, Zhuo-Yu Lu, Gui-Gang Zhao, Wei Shi, Ru-Wen Wan, Na Zhu, Jie-Qiong Jin, Jing Che

Supplementary material 2 Genetic matrices Data type xls https://binary.pensoft.net/file/1756257This dataset is made available under the Open Database License (http://opendatacommons.org/licenses/odbl/1.0/). The Open Database License (ODbL) is a license agreement intended to allow users to freely share, modify, and use this Dataset while maintaining this same freedom for others, provided that the original source and author(s) are credited. Kai Wang, Zhuo-Yu Lu, Gui-Gang Zhao, Wei Shi, Ru-Wen Wan, Na Zhu, Jie-Qiong Jin, Jing Che
